# Increasing COVID-19 Testing and Vaccination Uptake in the Take Care Texas Community-Based Randomized Trial: Adaptive Geospatial Analysis

**DOI:** 10.2196/62802

**Published:** 2025-02-11

**Authors:** Kehe Zhang, Jocelyn V Hunyadi, Marcia C de Oliveira Otto, Miryoung Lee, Zitong Zhang, Ryan Ramphul, Jose-Miguel Yamal, Ashraf Yaseen, Alanna C Morrison, Shreela Sharma, Mohammad Hossein Rahbar, Xu Zhang, Stephen Linder, Dritana Marko, Rachel White Roy, Deborah Banerjee, Esmeralda Guajardo, Michelle Crum, Belinda Reininger, Maria E Fernandez, Cici Bauer

**Affiliations:** 1 Department of Biostatistics and Data Science, School of Public Health, The University of Texas Health Science Center at Houston, 1200 Pressler St., RAS-E819, Houston, TX, 77030, United States, 1 7135009581; 2 Center for Spatial-Temporal Modeling for Applications in Population Sciences, School of Public Health, The University of Texas Health Science Center at Houston, Houston, TX, United States; 3 Department of Epidemiology, School of Public Health, The University of Texas Health Science Center at Houston, Houston, TX, United States; 4 Department of Epidemiology, School of Public Health, The University of Texas Health Science Center at Houston, Brownsville, TX, United States; 5 Center for Health Equity, School of Public Health, The University of Texas Health Science Center at Houston, Houston, TX, United States; 6 Biostatistics/Epidemiology/Research Design Component, Center for Clinical and Translational Sciences, The University of Texas Health Science Center at Houston, Houston, TX, United States; 7 Department of Internal Medicine, Division of Clinical and Translational Sciences, McGovern Medical School, The University of Texas Health Science Center at Houston, Houston, TX, United States; 8 Department of Management, Policy and Community Health, School of Public Health, The University of Texas Health Science Center at Houston, Houston, TX, United States; 9 Department of Quantitative and Qualitative Health Sciences, University of Texas School of Public Health San Antonio, San Antonio, TX, United States; 10 Harris County Public Health, Houston, TX, United States; 11 Houston Health Department, Houston, TX, United States; 12 Cameron County Public Health, San Benito, TX, United States; 13 Department of Preventive Medicine and Population Health, School of Medicine, The University of Texas at Tyler, Tyler, TX, United States; 14 Department of Health Promotion and Behavior Sciences, School of Public Health, The University of Texas Health Science Center at Houston, Houston, TX, United States

**Keywords:** COVID-19 testing, COVID-19 vaccination, study design, community-based interventions, geospatial analysis, public health, social determinants of health, data dashboard

## Abstract

**Background:**

Geospatial data science can be a powerful tool to aid the design, reach, efficiency, and impact of community-based intervention trials. The project titled *Take Care Texas* aims to develop and test an adaptive, multilevel, community-based intervention to increase COVID-19 testing and vaccination uptake among vulnerable populations in 3 Texas regions: Harris County, Cameron County, and Northeast Texas.

**Objective:**

We aimed to develop a novel procedure for adaptive selections of census block groups (CBGs) to include in the community-based randomized trial for the Take Care Texas project.

**Methods:**

CBG selection was conducted across 3 Texas regions over a 17-month period (May 2021 to October 2022). We developed persistent and recent COVID-19 burden metrics, using real-time SARS-CoV-2 monitoring data to capture dynamic infection patterns. To identify vulnerable populations, we also developed a CBG-level community disparity index, using 12 contextual social determinants of health (SDOH) measures from US census data. In each adaptive round, we determined the priority CBGs based on their COVID-19 burden and disparity index, ensuring geographic separation to minimize intervention “spillover.” Community input and feedback from local partners and health workers further refined the selection. The selected CBGs were then randomized into 2 intervention arms—multilevel intervention and just-in-time adaptive intervention—and 1 control arm, using covariate adaptive randomization, at a 1:1:1 ratio. We developed interactive data dashboards, which included maps displaying the locations of selected CBGs and community-level information, to inform the selection process and guide intervention delivery. Selection and randomization occurred across 10 adaptive rounds.

**Results:**

A total of 120 CBGs were selected and followed the stepped planning and interventions, with 60 in Harris County, 30 in Cameron County, and 30 in Northeast Texas counties. COVID-19 burden presented substantial temporal changes and local variations across CBGs. COVID-19 burden and community disparity exhibited some common geographical patterns but also displayed distinct variations, particularly at different time points throughout this study. This underscores the importance of incorporating both real-time monitoring data and contextual SDOH in the selection process.

**Conclusions:**

The novel procedure integrated real-time monitoring data and geospatial data science to enhance the design and adaptive delivery of a community-based randomized trial. Adaptive selection effectively prioritized the most in-need communities and allowed for a rigorous evaluation of community-based interventions in a multilevel trial. This methodology has broad applicability and can be adapted to other public health intervention and prevention programs, providing a powerful tool for improving population health and addressing health disparities.

## Introduction

The COVID-19 pandemic created a public health crisis, prompting discussions on how to best mitigate its far-reaching effects in a timely and efficient manner. From the start of the pandemic (March 2020) to May 2021 (the beginning of this study), there were over 30 million cumulative COVID-19 cases [1], over 2 million hospitalizations, and over half a million deaths in the United States alone [2]. During natural and health-related disasters (eg, pandemics and epidemics), vulnerable populations have historically endured a disproportionate level of negative health outcomes [3-7]. Extensive research has revealed similar impacts of the COVID-19 pandemic on vulnerable populations, particularly older adults, racial and ethnic minority groups, individuals who are immunocompromised, those with chronic conditions, and individuals with lower socioeconomic status and education attainment [8-17]. For example, a higher risk of severe COVID-19 symptoms, hospitalization, and mortality is associated with the presence of underlying medical conditions and older age [8,9]. Further, multiple studies have assessed population vulnerability through the Centers for Disease Control and Prevention (CDC) Social Vulnerability Index (SVI). For example, COVID-19 risk increases with the overall SVI score and the scores for specific SVI domains, notably those for the minority status and language domains [10,11,16]. Specifically, Hispanic and Black individuals have experienced higher rates of infection, hospitalization, and mortality when compared to non-Hispanic White individuals [12-14,18]. Racial and ethnic minority groups tend to be more susceptible to COVID-19, partially due to economic and social factors such as limited financial resources; insecure housing; higher housing density; reliance on public transportation; and additional inequities in nutrition, jobs, environments, and access to health care and health-related resources [13,14,19,20]. These findings underscore the importance and complex interplay of social determinants of health (SDOH) with respect to COVID-19 outcomes.

Health disparities research has consistently shown that geography—where individuals live, work, and play—matters, and it can greatly influence mortality, morbidity, life expectancy, and various aspects of health [21]. Geographic disparities in COVID-19 health outcomes have also been well documented. Previous studies have demonstrated county-level disparities in SARS-CoV-2 testing, infection, and fatality rates [22-24]. Research conducted by Das et al [25] reported geographic disparities in COVID-19 incidence across various zip code tabulation areas (ZCTAs) in the greater St. Louis area, Missouri. Das et al [25] also reported that the strength of the associations between the area proportion of the population working in agriculture and COVID-19 incidence varied by different ZCTAs. In a separate study, Bauer et al [26] developed a novel approach to identifying testing disparities by census block groups (CBGs), revealing significant geographical disparities in the context of smaller areas. Further, geographical disparities may not be constant and instead shift over time. For example, from 2019 to 2020, temporal trends in confirmed COVID-19 incidence and mortality differed between the most and least vulnerable counties in the United States [27]. Another study reported significant changes in the spatial-temporal patterns of the health care accessibility, test positivity rate of confirmed COVID-19 cases, and case fatality ratio in Texas [28].

Effective and real-time identification of vulnerable, at-risk populations that are characterized by diverse characteristics or reside within specific geographical regions is essential to address and eliminate disparities in COVID-19 health outcomes. In the context of health care, where resources are often limited and must be efficiently and equitably allocated, questions regarding site or population selection become paramount. Achieving this goal requires (1) the identification of metrics to accurately measure the multifaceted nature of disparities with available datasets and (2) a robust method to summarize all metrics and provide a meaningful output with practical use. The latter has been achieved through various approaches, such as assigning weights to each metric, which are often provided by domain experts, and constructing an index from the weighted sum of all metrics.

This paper presents an innovative protocol for adaptive geospatial selections in a community-based intervention program—the Take Care Texas project—funded by the National Institutes of Health (NIH) Rapid Access to Diagnostics for Underserved Populations (RADx-UP) program. Our protocol leveraged a data-driven approach for the rapid adaptation and deployment of multilevel, just-in-time adaptive interventions to improve COVID-19 testing and vaccination uptake in Texas. In the *Methods* section, we focus on 2 key areas. First, we outline the criteria and metrics for identifying the vulnerable populations via SDOH and geographical measures. Second, we detail the methodology for selecting the local communities based on CBGs, which involved using a phased, multistage approach. Designed for real-time prioritization, our protocol addresses the immediate needs of communities most affected by the changing landscape of the COVID-19 crisis.

## Methods

### Study Region and Populations

Our Take Care Texas project includes 3 geographically distinct sites within Texas, each with unique demographic characteristics (Multimedia Appendix 1). Southeast Texas, represented by the Houston area within Harris County, has an ethnically diverse population (N=4.73 million), covering both urban and suburban communities. This region serves as a significant residence for various racial and ethnic minority groups and is recognized for having one of the largest metropolitan economies in the United States. The South Texas site, represented by Cameron County (population: N=423,029) in the Rio Grande Valley, is primarily Hispanic, with a considerable proportion of the population (22.6%) living below the federal poverty line, exceeding the national average (11.5%) [29]. The Northeast Texas (NETX) region covers an extensive area, including the Smith, Gregg, Henderson, Van Zandt, Anderson, Wood, and Rains counties. NETX is characterized by a mix of small cities and rural communities, with a lower population density when compared to other sites. Anderson County was excluded from this study due to insufficient SARS-CoV-2 infection data coverage at the time of analysis. The demographic distribution across these regions reflects a wide socioeconomic and cultural spectrum, offering a comprehensive understanding of the diverse population within Texas. There are 2144 CBGs in Harris County, 222 CBGs in Cameron County, and 349 CBGs from the NETX region, according to the 2010 census data.

### Overview of Priority CBG Selection Process

**Figure 1.**
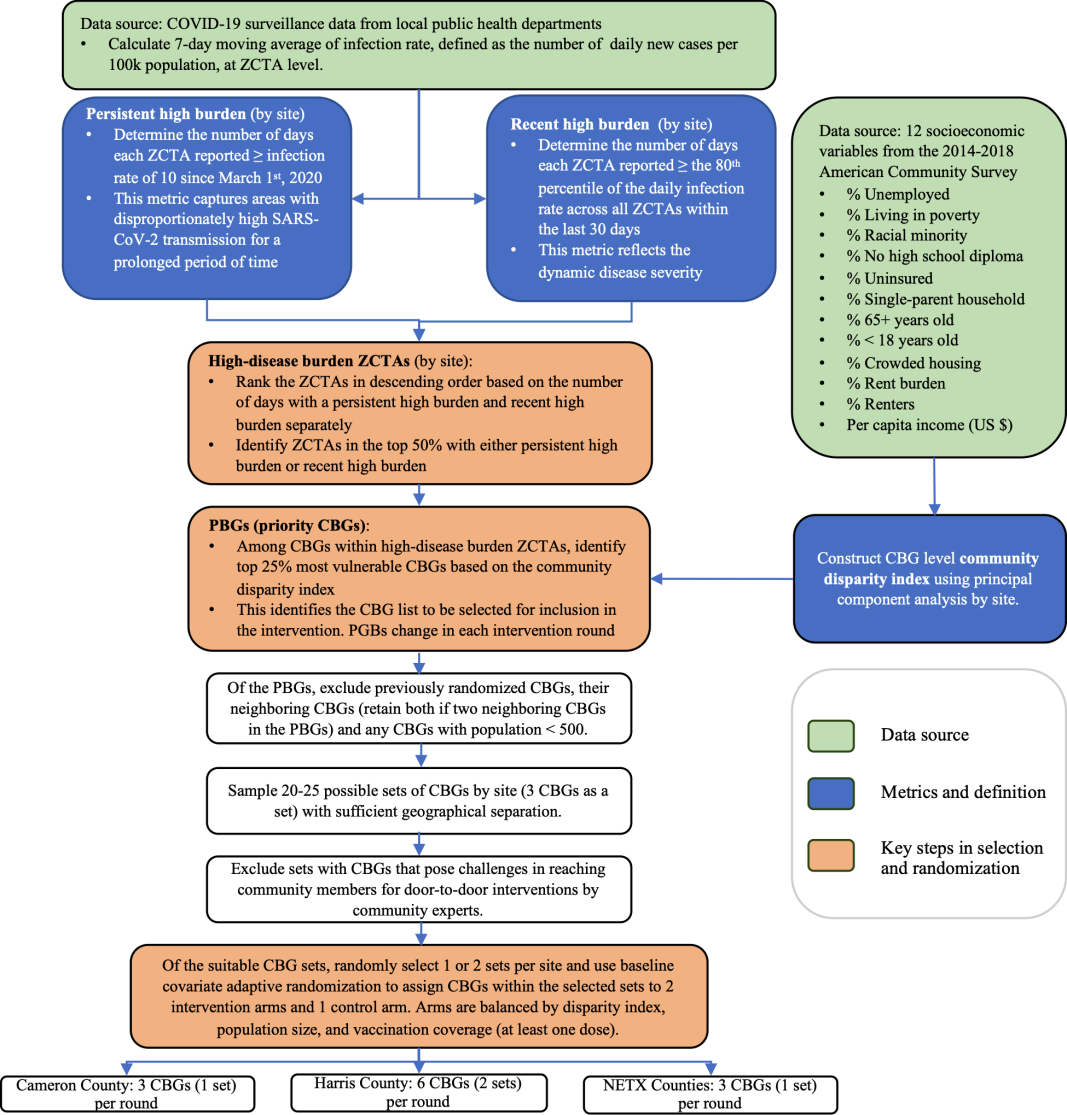
Workflow of the adaptive geospatial selection of CBGs for intervention in the Take Care Texas study. The selection of CBGs for intervention was conducted each round. CBG: census block group; NETX: Northeast Texas; PBG: priority census block group; ZCTA: zip code tabulation area.

The process for identifying priority CBGs (PBGs) in this study is presented in Figure 1. The process began with the identification of geographic areas experiencing significant COVID-19 burden, using SARS-CoV-2 surveillance data and population estimates at the ZCTA level. We then identified CBGs within these areas that exhibited high socioeconomic disparities, using the community disparity index we constructed to achieve the desired spatial granularity. Detailed descriptions for determining disease burden and community disparities are provided in the *Persistent and Recent COVID-19 Burdens* and *CBG-Level Community Disparity Index* sections.

In response to the dynamic nature of the COVID-19 pandemic, our identification and selection process was conducted monthly with a near–real-time data feed from the local public health department for timely and effective intervention planning. Table 1 illustrates the stepped planning and intervention calendar. Between May 2021 and October 2022, a total of 10 rounds of CBG selection were conducted for each of the three study sites. In each round, among the CBGs within the identified high–disease burden ZCTAs, we selected the most vulnerable CBGs based on the community disparity index (ie, CBGs in the top 25% for this metric). We then excluded previously randomized CBGs, their neighbors (ie, CBGs that share a common geographic boundary), and any CBGs with small populations (total population<500) to form the PBG list, which served as the sampling universe. From the PBGs, we sampled geographically nonadjacent sets of CBGs (3 CBGs as a set), ensuring suitable geographic separation to mitigate potential intervention “spillover,” since the study goal was to compare 2 distinct intervention strategies to a control arm. We sought feedback from the community health workers and staff in the three regions, to determine if the potential CBGs presented safety, geography, and access concerns, by showing maps of the areas and then discussing the areas and documenting concerns. When there was lack of clarity about an area, we reached out via phone calls and emails to community experts in our advisory groups (health department leaders, city leaders, and local nonprofits serving the areas) to obtain their feedback as well. CBGs that posed challenges in reaching community members (such as CBGs with primarily gated communities or business districts), which would prevent implementing the intervention protocol (eg, door-to-door visits), were excluded from the sampling frame. This collaborative approach ensured that our selection process was informed by both data-driven analysis and community and implementer insights. The final selected CBGs within each set were then assigned to 1 of 2 intervention groups (multilevel intervention and just-in-time adaptive intervention) or a control group through baseline covariate adaptive randomization, balancing disparity index and population size, as well as vaccination coverage when such data were available. These CBGs then underwent a 1-month planning phase, followed by a 2-month intervention period. In each round, this study selected and randomized 6 CBGs (2 sets) for Harris County due to its larger geographic area and population, 3 CBGs for Cameron County, and 3 CBGs for NETX counties.

**Table 1.** The stepped planning and intervention calendar by month. Each round, selected census block groups were randomized into 1 control arm and 2 intervention arms—multilevel intervention (MILI) and just-in-time adaptive intervention (JITAI).

		2021								2022					
		May	June	July	August	September	October	November	December	January	February	March	April	May	June
Round 1															
	Control	✓^a^	✓^b^	✓^b^											
	MILI	✓^a^	✓^b^	✓^b^											
	JITAI	✓^a^	✓^b^	✓^b^											
Round 2															
	Control		✓^a^	✓^b^	✓^b^										
	MILI		✓^a^	✓^b^	✓^b^										
	JITAI		✓^a^	✓^b^	✓^b^										
Round 3															
	Control			✓^a^	✓^b^	✓^b^									
	MILI			✓^a^	✓^b^	✓^b^									
	JITAI			✓^a^	✓^b^	✓^b^									
Round 4															
	Control				✓^a^	✓^b^	✓^b^								
	MILI				✓^a^	✓^b^	✓^b^								
	JITAI				✓^a^	✓^b^	✓^b^								
Round 5															
	Control					✓^a^	✓^b^	✓^b^							
	MILI					✓^a^	✓^b^	✓^b^							
	JITAI					✓^a^	✓^b^	✓^b^							
Round 6															
	Control						✓^a^	✓^b^	✓^b^						
	MILI						✓^a^	✓^b^	✓^b^						
	JITAI						✓^a^	✓^b^	✓^b^						
Round 7															
	Control									✓^a^	✓^b^	✓^b^			
	MILI									✓^a^	✓^b^	✓^b^			
	JITAI									✓^a^	✓^b^	✓^b^			
Round 8															
	Control										✓^a^	✓^b^	✓^b^		
	MILI										✓^a^	✓^b^	✓^b^		
	JITAI										✓^a^	✓^b^	✓^b^		
Round 9															
	Control											✓^a^	✓^b^	✓^b^	
	MILI											✓^a^	✓^b^	✓^b^	
	JITAI											✓^a^	✓^b^	✓^b^	
Round 10															
	Control												✓^a^	✓^b^	✓^b^
	MILI												✓^a^	✓^b^	✓^b^
	JITAI												✓^a^	✓^b^	✓^b^

a Planning months.

b Intervention months.

### Persistent and Recent COVID-19 Burdens

The persistent and recent high COVID-19 burdens were quantified by using SARS-CoV-2 surveillance data obtained from local public health departments. We computed a 7-day moving average for infection rate, which was defined as the daily new cases per 100,000 population. For each ZCTA, the persistent disease burden was evaluated based on the number of days on which ≥10 new cases per 100,000 population were reported, as per the common COVID-19 risk level threshold [30], starting from March 1, 2020, up to the respective analysis date for each selection round. To evaluate the recent disease burden, we first calculated the 80th percentile of daily new cases per 100,000 population across all ZCTAs within each study site, using data from the 30 days preceding each analysis date. We then counted the number of days on which the infection rate for each ZCTA exceeded this 80th percentile threshold. In each selection round and for each site, we ranked the ZCTAs, in descending order, based on their number of days with (1) a persistent high burden and (2) a recent high burden. The ranking was performed for each metric separately, and the ZCTAs in the top 50% for either metric were identified as high–disease burden ZCTAs.

### CBG-Level Community Disparity Index

We developed the CBG-level community disparity index due to the lack of spatial granularity in existing indices, such as the SVI, which did not meet our study’s needs. Using 12 SDOH measures from the 2014‐2018 American Community Survey 5-year estimates [31], our community disparity index covered the socioeconomic, demographic, and housing dimensions of SDOH (Multimedia Appendix 2). It integrated economic stability factors (employment and poverty rates, per capita income, education level, and health insurance coverage), demographic characteristics (percentages of individuals younger than 18 years, individuals older than 65 years, individuals in single-parent households, and racial minority individuals), and housing factors (percentage of renter-occupied units, rent burden, and crowded housing).

We used principal component analysis (PCA) to construct the community disparity index, similar to the approach used by Kolak et al [32]. PCA is a data-driven approach that synthesizes multiple pieces of information into one combined measure, while simultaneously handling high correlation and minimizing information loss [32-35]. We kept the principal components with eigenvalues exceeding 1, following the Kaiser criteria. The scores of these selected principal components were weighted according to the variance they accounted for and then summed, with higher scores representing a greater community disparity. The resulting indices were then ranked and scaled from 0 to 1 within each study region, providing a relative ranking of CBGs. All data were processed and analyzed with R statistical software (v4.1.2; R Foundation for Statistical Computing) [36].

### Interactive Data Dashboards

We developed ArcGIS (Environmental Systems Research Institute Inc) and R Shiny (Posit PBC) data dashboards that provided key geospatial information and relevant COVID-19 data for this study. Feedback was received from the dashboard’s end users, including community health workers. The dashboards included maps that showed community disparity indices, PBGs, intervention-prioritized CBGs, COVID-19 testing sites, road maps, and SARS-CoV-2 infection and testing rates at the CBG and ZCTA levels. These tools enabled community health workers to identify the boundaries of the priority areas and access the relevant COVID-19 data for effective on-ground intervention messaging and referral to needed services. Both dashboards were designed to be interactive to facilitate strategic planning efforts and were password protected for internal team use only. The dashboard was especially useful during the planning phase and facilitated the creation of a community profile document, which community health workers used to share real-time data and deliver messaging tailored to the communities.

### Ethical Considerations

The research protocol was approved by the UTHealth Committee for the Protection of Human Subjects (HSC-SPH-20‐1372). Data user agreements were established between UTHealth and the public health departments of Harris County, Cameron County, and NETX counties. This study did not involve direct contact with participants, as data were aggregated at the CBG level, so no individual-level informed consent was required. Data were deidentified, and all analyses followed data privacy guidelines.

## Results

Table 2 presents summary statistics of the population density and 12 SDOH variables used to construct the community disparity index for CBGs in Harris County, Cameron County, and NETX counties, in comparison to the whole state of Texas. Harris County, with the highest population density (2600 per km^2^) and per capita income (US $33,500), also has the highest percentage of renters (44.9%). Cameron County, on the other hand, has a higher percentage of crowded housing (11.2%), individuals without high school diplomas (35.1%), uninsured individuals (28.0%), people living in poverty (30.9%), and racial minority individuals (90.0%). NETX has a higher percentage of people aged 65 years and older (18.2%) but a much lower proportion of racial minority individuals compared to Texas overall (35% vs 57.7%).

Between March 2021 and March 2023, we identified 5 distinct COVID-19 waves, with 3 occurring within the intervention period of our study, specifically around August 2021, December 2021, and July 2022 (Multimedia Appendix 3). The overall infection rates varied across the three study regions, highlighting the importance of site-specific burden assessment. The temporal trends of SARS-CoV-2 infection rates differed among the sites when the pandemic started but became more similar during the intervention period, that is, from May 2021 to October 2022. Harris County consistently reported higher infection rates than those reported by the other two regions throughout this study.

**Figure 2.**
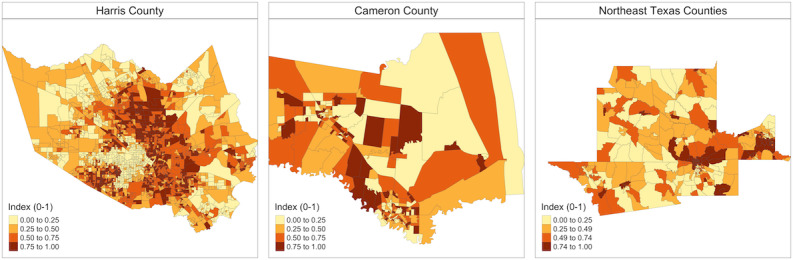
Census block group–level community disparity index developed for the community-based intervention by study region. The community disparity index was constructed by using principal component analysis to combine 12 social determinants of health measures from the American Community Survey 2014-2018 5-year estimates [31]. The index was scaled to range from 0 to 1 within each study area, with higher index values indicating greater community disparity.

Figure 2 displays maps of the CBG-level community disparity index, highlighting substantial variations across and within the study regions. The leading four PCA components captured a significant portion of the variance in the 12 SDOH measures. For instance, in Harris County, these components explained 70.96% of the variance, with the first principal component alone accounting for 43.45%, and were primarily influenced by socioeconomic factors (percentage of individuals with no high school diploma, percentage of individuals living under poverty, per capita income, percentage of uninsured individuals, and percentage of racial minority individuals). Consequently, we derived the standardized index scores by using the first four PCA components.

The maps of the COVID-19 burden and community disparity index shared similar geographic patterns, with high COVID-19 burdens often found in ZCTAs with elevated community disparity (Multimedia Appendix 4). However, distinctive patterns also existed. For instance, during the first selection round for Harris County in May 2021, several ZCTAs located in the western and eastern parts of the county experienced high COVID-19 burden, despite their low community disparity scores. This highlights the complex interplay between the pandemic impact and SDOH, as COVID-19 can affect diverse communities, including those traditionally considered less socially vulnerable. Therefore, it is crucial to leverage both SDOH data and COVID-19 surveillance data to dynamically identify and prioritize areas for interventions.

**Figure 3.**
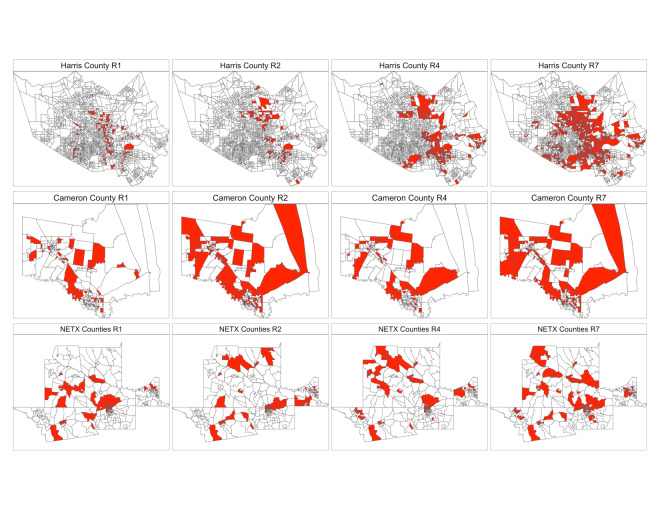
Priority CBGs for the community-based intervention by study region and selection rounds: highlighted CBGs were identified as candidates for inclusion in R1, R2, R4, and R7. CBG: census block group; NETX: Northeast Texas; R: round.

Over the 17-month period from May 2021 to October 2022, we selected and randomized 120 CBGs for intervention across 10 rounds, with 60 CBGs in Harris County, 30 in Cameron County, and 30 in NETX counties (Table 1). The population size of the randomized CBGs ranged from 615 to 11,321. The combination of the community disparity index and real-time COVID-19 surveillance data allowed for the identification and selection of areas—those with the highest disease burden among the most vulnerable populations—for the adaptive, multilevel, just-in-time intervention. This study also observed substantial changes in the PBG list across different rounds, with more CBGs identified in the later rounds of selection, corresponding to increased infection rates during the pandemic, as shown in Figure 3.

Multimedia Appendix 5 illustrates the R Shiny and ArcGIS dashboards that we developed to provide a dynamic visualization of the data from this study. These interactive dashboards, along with the community profiles we developed, were used by the intervention team to tailor their strategies. By leveraging these tools, the team was able to adapt culturally relevant messages and materials, enhance door-to-door education by community health workers in selected CBGs, and better inform social marketing campaigns to promote COVID-19 testing and vaccination efforts.

**Table 2.** Summary statistics of the American Community Survey variables (2014-2018 5-year estimates) [31] used in community disparity index creation at the census block group (CBG) level for 3 regions in Texas and Texas overall.

	Texas regions			Texas overall
	Cameron County (CBGs: n=222)	Harris County (CBGs: n=2144)	NETX^a^ counties (CBGs: n=349)	
Population (n)				27,900,000
Mean (CV^b^ [%])	1900 (83.7)	2150 (83.5)	1540 (48.6)	—^c^
Median (IQR)	1550 (967-2350)	1770 (1210-2470)	1370 (1060-1890)	—
Population density (per km^2^)				43.1
Mean (SD [%])	1420 (86.5)	2600 (97.5)	546 (136.5)	—
Median (minimum, maximum)	1180 (345, 2130)	2020 (1180, 3090)	209 (41.4, 870)	—
Crowded housing (%)				4.79
Mean (CV [%])	11.2 (74.0)	6.28 (123.8)	3.45 (141.4)	—
Median (IQR)	9.84 (4.82-16.3)	3.68 (0-9.46)	1.82 (0-5.24)	—
Individuals without a high school diploma (%)				16.8
Mean (CV [%])	35.1 (45.1)	21.5 (84.1)	16.7 (74.3)	—
Median (IQR)	36.0 (23.2-47.8)	17.0 (5.65-34.5)	14.1 (7.93-21.5)	—
Renters (%)				38.1
Mean (CV [%])	36.0 (56.4)	44.9 (65.1)	32.2 (64.9)	—
Median (IQR)	34.0 (20.9-46.1)	40.2 (20.5-66.5)	27.4 (17.2-43.7)	—
Rent burden (%)				44.4
Mean (CV [%])	46.8 (45.9)	43.7 (52.2)	39.7 (61.6)	—
Median (IQR)	46.4 (33.3-60.9)	45.5 (29.0-58.2)	39.1 (22.2-56.3)	—
Individuals younger than 18 years (%)				26.2
Mean (CV [%])	29.1 (29.6)	25.3 (36.2)	23.7 (36.4)	—
Median (IQR)	30.1 (23.6-35.4)	25.8 (19.5-31.5)	23.0 (18.2-29.0)	—
Individuals older than 65 years (%)				12.0
Mean (CV [%])	15.5 (69.0)	11.3 (66.7)	18.2 (53.7)	—
Median (IQR)	13.2 (9.67-18.4)	9.99 (6.28-14.5)	17.2 (11.0-23.5)	—
Individuals in single-parent households (%)				14.7
Mean (CV [%])	18.3 (62.5)	17.2 (83.3)	14.0 (92.8)	—
Median (IQR)	17.1 (9.94-25.8)	14.4 (6.23-25.3)	10.8 (4.72-20.9)	—
Unemployed individuals (%)				5.41
Mean (CV [%])	7.05 (91.1)	6.45 (91.4)	5.65 (103.5)	—
Median (IQR)	5.85 (2.60-9.76)	5.16 (2.54-8.89)	4.39 (1.47-7.87)	—
Per capita income (US $)				30,100
Mean (CV [%])	16,400 (45.3)	33,500 (78.2)	25,800 (41.8)	—
Median (IQR)	14,500 (10,700-20,200)	24,200 (16,700-40,700)	25,400 (17,600-30,700)	—
Uninsured individuals (%)				17.1
Mean (CV [%])	28.0 (38.4)	20.9 (67.3)	17.6 (54.8)	—
Median (IQR)	28.4 (20.2-35.3)	19.5 (9.25-30.9)	16.0 (10.6-23.4)	—
Individuals living in poverty (%)				14.3
Mean (CV [%])	30.9 (46.9)	16.2 (81.2)	16.6 (71.9)	—
Median (IQR)	31.1 (19.1-42.1)	13.2 (5.63-23.9)	14.5 (7.93-22.4)	—
Racial minority individuals (%)				57.7
Mean (CV [%])	90.0 (15.6)	69.3 (40.0)	35.0 (77.2)	—
Median (IQR)	94.2 (87.3-98.4)	79.7 (46.5-94.0)	26.1 (14.4-50.9)	—

a NETX: Northeast Texas.

b CV: coefficient of variation.

c Not applicable.

## Discussion

### Principal Findings

In this study, we used geospatial data science methods to develop a protocol for the adaptive selection of 120 CBGs in 3 regions of Texas, which occurred across 10 rounds over a 17-month period (May 2021 to October 2022). This protocol, which was developed for the Take Care Texas project, integrated real-time dynamic data on SARS-CoV-2 infection rates and contextual SDOH to identify and prioritize the CBGs most in need of tailored activities and resources to improve COVID-19 testing and vaccination. The adaptive selection of CBGs prioritized the most vulnerable communities and allowed for the rigorous evaluation of community-based interventions in a multilevel trial.

### Strengths and Limitations

Our protocol demonstrated several unique strengths in integrating public health surveillance data and geospatial data science. First, our study offered spatial granularity at the level of CBGs, which has rarely been seen in other studies [11,16,37]. Such granularity could potentially result in more tailored interventions that are best suited to the local communities for which they were developed. Second, we incorporated both the reported SARS-CoV-2 infection rates and the community SDOH, reflecting the socioeconomic and housing conditions that contributed to health disparities. This allowed us to prioritize communities experiencing not only high disease burden but also high social vulnerability that could potentially impact community resilience and the ability to adapt to challenges presented by the pandemic. Third, the dynamic nature of our protocol, particularly the initial selection of high–disease burden ZCTAs, allowed for real-time adaptation of the selection strategies in response to the changing pandemic, thereby enabling us to prioritize the most in-need and most vulnerable communities effectively. Finally, the protocol was strengthened by the input from and collaboration among community members and frontline community health workers, which were critical in designing and implementing interventions. The final CBG selection was made by community partners considering on-the-ground intervention feasibility. In the context of this study and site selection, the input from our community partners and community health workers supplemented the data-driven approaches [38]. Although multicriteria decision-making methods have been previously used for site selection problems [39-42], including the integration of objective criteria with experts’ qualitative input to determine site suitability, some multicriteria decision-making techniques have been critiqued for arbitrary and irrational rankings of alternatives [43] and questionable mathematical validity [44]. By contrast, previous work has shown that PCA is a robust approach for integrating various vulnerability and health indicators and providing reliable unequal weighting [33,34] to help guide the selection of local communities for health care interventions. Future studies should continue to investigate best practices for engaging communities in trial design and implementation.

Our protocol also presented several limitations. First, the COVID-19 pandemic highlighted significant challenges in data reporting due to the absence of established surveillance data infrastructures for COVID-19 and other rapidly spreading communicable diseases. The state reporting requirements for data elements and definitions changed over time, complicating the consistent use of data across study regions. In our study, we directly collaborated with individual county public health departments and processed and harmonized data across different study regions, which were time-consuming processes. Second, the time lag commonly seen in surveillance databases posed challenges in accurately identifying current hot spots, and incomplete demographic data hindered the assessment of health equity throughout the COVID-19 pandemic by delaying the analysis of COVID-19 health outcomes by subgroups and across more granular geographic scales [45,46]. The Congressional Research Service’s 2020 report highlighted the need to modernize public health reporting systems to improve the timeliness and accuracy of public health data reporting [45]. However, differences in data standards, technical capacity, and regional policy priorities, particularly in the early stages of the COVID-19 pandemic, limited effective data sharing across study regions. Third, our protocol required accurate and complete address information for geographical granularity in identifying the high–disease burden priority areas, and when address information was missing, we may not have been able to include the SARS-CoV-2 case records in determining the PBGs. Finally, finding geographically separated CBGs in later rounds of selection could be difficult in small counties with fewer CBGs, such as Cameron County. Traditionally, administrative boundaries are used to define spatial units, yet alternative approaches may better capture actual community layouts. For example, Tuson et al [47] developed the Spatial Targeting Algorithm to generate flexible priority regions not restricted by administrative boundaries. However, while flexible boundaries may better capture neighborhoods, they may not provide SDOH information, which is typically accessible only at the administrative level. Additional work is needed to develop more flexible spatial units, with or without administrative boundaries as a constraint, for population-based research.

### Study Implications

Despite these challenges, our study provided valuable protocols for data processing and analytical support to an intervention team working with local public health departments and organizations. It demonstrated a useful approach to ensuring that the most vulnerable communities participated in a multilevel intervention trial for increasing COVID-19 testing and vaccination uptake. Our proposed protocol could be adapted to other population-based randomized trials for which rapid assessment and allocation of finite resources are imperative. For example, geospatial approaches have been used to assess cancer screening rates among a vulnerable population in Ontario, Canada [48] and determine priority clinics to allocate funding for improving up-to-date colorectal cancer screening rates [49]. The integration of PCA and the real-time nature of our protocol can also be adapted to other population-based trials. When tackling multifaceted and complex problems, our protocol demonstrates that PCA is effective at robustly incorporating a wide range of health indicators. Further, the real-time nature of our protocol allows for ongoing, continuous assessment and adjustment in allocation of resources, provided that accurate and timely data are available. This could be applied to study recruitment protocols or other intervention-based trials.

### Conclusion

The selection of optimal sites for health care and public health interventions, especially under limited resources, is a crucial challenge. Our protocol contributes to existing research by integrating disease surveillance and SDOH data, the robust PCA method, and community engagement to identify locations where COVID-19 testing interventions for vulnerable populations are most needed. The proposed data-driven approach could be adapted to other disease control and prevention programs to improve population health and reduce disease burden in areas with disparities.

## Supplementary material

10.2196/62802 Multimedia Appendix 1 Map of Texas counties with 3 study regions highlighted. Harris County (yellow) has the most ethnically diverse population in Texas. Cameron County (blue), located on the southern border of Texas, is primarily Hispanic. A large proportion of the population in Northeast Texas (NETX; orange) is White.

10.2196/62802 Multimedia Appendix 2 Metrics for identifying vulnerable populations, definitions, data sources, and available spatial scale.

10.2196/62802 Multimedia Appendix 3 Temporal trends of weekly COVID-19 cases per 100,000 population in Harris County, Cameron County, and Northeast Texas (NETX) counties, with vertical lines indicating the start and end of the planning and intervention periods for the Take Care Texas project. Historical Texas COVID-19 data were obtained from the Texas Department of State Health Services.

10.2196/62802 Multimedia Appendix 4 Maps of the census block group–level community disparity index developed in this protocol, overlaid with high–disease burden zip code tabulation areas (shown in red geographical boundaries) in the first round of selection (May 2021) for the three study regions.

10.2196/62802 Multimedia Appendix 5 Screenshots of the (A) R shiny dashboard and (B) ArcGIS dashboard developed for the Take Care Texas study. These data dashboards were designed to be interactive to best facilitate strategic planning efforts and were password protected for internal team use only.

## Abbreviations

CBG: census block group
CDC: Centers for Disease Control and Prevention
NETX: Northeast Texas
NIH: National Institutes of Health
PBG: priority census block group
PCA: principal component analysis
RADx-UP: Rapid Access to Diagnostics for Underserved Populations
SDOH: social determinants of health
SVI: Social Vulnerability Index
ZCTA: zip code tabulation area

## References

[1] WHO COVID-19 dashboard. World Health Organization.

[2] COVID data tracker. Centers for Disease Control and Prevention.

[3] Paramanathan P, Abbas M, Huda SA, Huda S, Mortazavi M, Taravati P (2021). Comparing racial health disparities in pandemics a decade apart: H1N1 and COVID-19. Future Healthc J.

[4] Quinn SC, Kumar S, Freimuth VS, Musa D, Casteneda-Angarita N, Kidwell K (2011). Racial disparities in exposure, susceptibility, and access to health care in the US H1N1 influenza pandemic. Am J Public Health.

[5] D’Adamo A, Schnake-Mahl A, Mullachery PH, Lazo M, Diez Roux AV, Bilal U (2023). Health disparities in past influenza pandemics: a scoping review of the literature. SSM Popul Health.

[6] Howell J, Elliott JR (2019). Damages done: the longitudinal impacts of natural hazards on wealth inequality in the United States. Soc Probl.

[7] (2021). Climate change and social vulnerability in the United States: a focus on six impacts. United States Environmental Protection Agency.

[8] Sanyaolu A, Okorie C, Marinkovic A (2020). Comorbidity and its impact on patients with COVID-19. SN Compr Clin Med.

[9] Souch JM, Cossman JS (2021). A commentary on rural-urban disparities in COVID-19 testing rates per 100,000 and risk factors. J Rural Health.

[10] Karaye IM, Horney JA (2020). The impact of social vulnerability on COVID-19 in the U.S.: an analysis of spatially varying relationships. Am J Prev Med.

[11] Khazanchi R, Beiter ER, Gondi S, Beckman AL, Bilinski A, Ganguli I (2020). County-level association of social vulnerability with COVID-19 cases and deaths in the USA. J Gen Intern Med.

[12] Millett GA, Jones AT, Benkeser D (2020). Assessing differential impacts of COVID-19 on black communities. Ann Epidemiol.

[13] Aleligne YK, Appiah D, Ebong IA (2021). Racial disparities in coronavirus disease 2019 (COVID-19) outcomes. Curr Opin Cardiol.

[14] Mackey K, Ayers CK, Kondo KK (2021). Racial and ethnic disparities in COVID-19-related infections, hospitalizations, and deaths : a systematic review. Ann Intern Med.

[15] Upshaw TL, Brown C, Smith R, Perri M, Ziegler C, Pinto AD (2021). Social determinants of COVID-19 incidence and outcomes: a rapid review. PLoS One.

[16] Biggs EN, Maloney PM, Rung AL, Peters ES, Robinson WT (2021). The relationship between social vulnerability and COVID-19 incidence among Louisiana census tracts. Front Public Health.

[17] Bauer C, Zhang K, Lee M (2021). Census tract patterns and contextual social determinants of health associated with COVID-19 in a Hispanic population from South Texas: a spatiotemporal perspective. JMIR Public Health Surveill.

[18] Tai DBG, Shah A, Doubeni CA, Sia IG, Wieland ML (2021). The disproportionate impact of COVID-19 on racial and ethnic minorities in the United States. Clin Infect Dis.

[19] Caraballo C, Ndumele CD, Roy B (2022). Trends in racial and ethnic disparities in barriers to timely medical care among adults in the US, 1999 to 2018. JAMA Health Forum.

[20] Radley DC, Baumgartner JC, Collins SR, Zephyrin LC, Schneider EC (2021). Achieving racial and ethnic equity in U.S. health care: a scorecard of state performance. The Commonwealth Fund.

[21] Baciu A, Negussie Y, Geller A, Weinstein JN, National Academies of Sciences, Engineering, and Medicine, Health and Medicine Division, Board on Population Health and Public Health Practice, Committee on Community-Based Solutions to Promote Health Equity in the United States (2017). Communities in Action: Pathways to Health Equity.

[22] Deb Nath N, Khan MM, Schmidt M, Njau G, Odoi A (2023). Geographic disparities and temporal changes of COVID-19 incidence risks in North Dakota, United States. BMC Public Health.

[23] Khan MM, Odoi A, Odoi EW (2023). Geographic disparities in COVID-19 testing and outcomes in Florida. BMC Public Health.

[24] Jackson SL, Derakhshan S, Blackwood L (2021). Spatial disparities of COVID-19 cases and fatalities in United States counties. Int J Environ Res Public Health.

[25] Das P, Igoe M, Lenhart S (2022). Geographic disparities and determinants of COVID-19 incidence risk in the greater St. Louis Area, Missouri (United States). PLoS One.

[26] Bauer C, Li X, Zhang K (2023). A novel Bayesian spatial-temporal approach to quantify SARS-CoV-2 testing disparities for small area estimation. Am J Public Health.

[27] Neelon B, Mutiso F, Mueller NT, Pearce JL, Benjamin-Neelon SE (2021). Spatial and temporal trends in social vulnerability and COVID-19 incidence and death rates in the United States. PLoS One.

[28] Park J, Michels A, Lyu F, Han SY, Wang S (2023). Daily changes in spatial accessibility to ICU beds and their relationship with the case-fatality ratio of COVID-19 in the state of Texas, USA. Appl Geogr.

[29] (2023). U.S. Census Bureau QuickFacts: Cameron County, Texas. United States Census Bureau.

[30] Harvard Global Health Institute, Edmond J. Safra Center for Ethics (2020). Key metrics for COVID suppression: a framework for policy makers and the public. Global Epidemics.

[31] 2014-2018 ACS 5-year estimates. United States Census Bureau.

[32] Kolak M, Bhatt J, Park YH, Padrón NA, Molefe A (2020). Quantification of neighborhood-level social determinants of health in the continental United States. JAMA Netw Open.

[33] Niu Y, Li Z, Gao Y (2021). A systematic review of the development and validation of the heat vulnerability index: major factors, methods, and spatial units. Curr Clim Change Rep.

[34] Pathak S, Wang H, Seals K, Adusumilli NC, Holston D (2023). Self-assessed health status and obesity vulnerability in rural Louisiana: a cross-sectional analysis. PLoS One.

[35] Lieberman-Cribbin W, Tuminello S, Flores RM, Taioli E (2020). Disparities in COVID-19 testing and positivity in New York City. Am J Prev Med.

[36] R Core Team (2022). R: a language and environment for statistical computing. R Foundation for Statistical Computing.

[37] Desjardins MR, Hohl A, Delmelle EM (2020). Rapid surveillance of COVID-19 in the United States using a prospective space-time scan statistic: detecting and evaluating emerging clusters. Appl Geogr.

[38] Cromley E, Kleinman LC, Ramos MA (2011). A community-engaged approach to select geographic areas for interventions to reduce health disparities. Prog Community Health Partnersh.

[39] Mardani A, Hooker RE, Ozkul S (2019). Application of decision making and fuzzy sets theory to evaluate the healthcare and medical problems: a review of three decades of research with recent developments. Expert Syst Appl.

[40] Boyacı AÇ, Şişman A (2022). Pandemic hospital site selection: a GIS-based MCDM approach employing Pythagorean fuzzy sets. Environ Sci Pollut Res Int.

[41] Tripathi AK, Agrawal S, Gupta RD (2022). Comparison of GIS-based AHP and fuzzy AHP methods for hospital site selection: a case study for Prayagraj City, India. GeoJournal.

[42] Sotoudeh-Anvari A (2022). The applications of MCDM methods in COVID-19 pandemic: a state of the art review. Appl Soft Comput.

[43] Asadabadi MR, Chang E, Saberi M (2019). Are MCDM methods useful? A critical review of Analytic Hierarchy Process (AHP) and Analytic Network Process (ANP). Cogent Engineering.

[44] Zhü K (2014). Fuzzy analytic hierarchy process: fallacy of the popular methods. Eur J Oper Res.

[45] Sekar K, Napili A (2020). Tracking COVID-19: U.S. public health surveillance and data. https://crsreports.congress.gov/product/pdf/R/R46588.

[46] Krieger N, Gonsalves G, Bassett MT, Hanage W, Krumholz HM (2020). The fierce urgency of now: closing glaring gaps in US surveillance data on COVID-19. Health Affairs.

[47] Tuson M, Yap M, Kok MR (2022). Improving the efficiency of geographic target regions for healthcare interventions. Int J Environ Res Public Health.

[48] Lofters AK, Gozdyra P, Lobb R (2013). Using geographic methods to inform cancer screening interventions for South Asians in Ontario, Canada. BMC Public Health.

[49] Zhan FB, Liu Y, Yang M (2023). Using GIS to identify priority sites for colorectal cancer screening programs in Texas health centers. Prev Chronic Dis.

