# Supplementary figures and images for: Increasing COVID-19 Testing and Vaccination Uptake in the Take Care Texas Community-Based Randomized Trial: Adaptive Geospatial Analysis
Source: JMIR Form Res. 2025 Feb 11;9:e62802. doi: 10.2196/62802 (PMC11835599; doi:10.2196/62802)

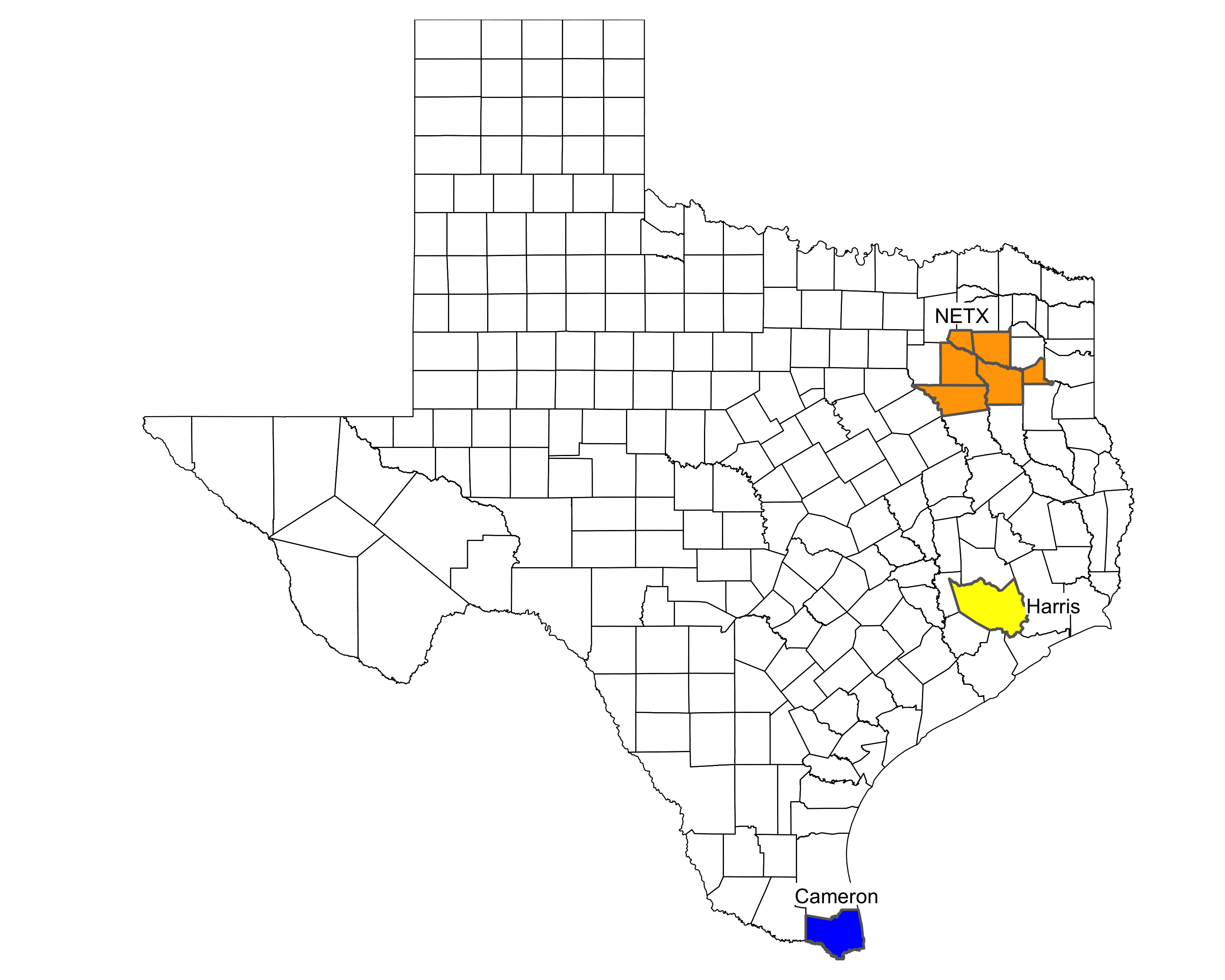

Supplement: Multimedia Appendix 1 [file formative-v9-e62802-s001.png]

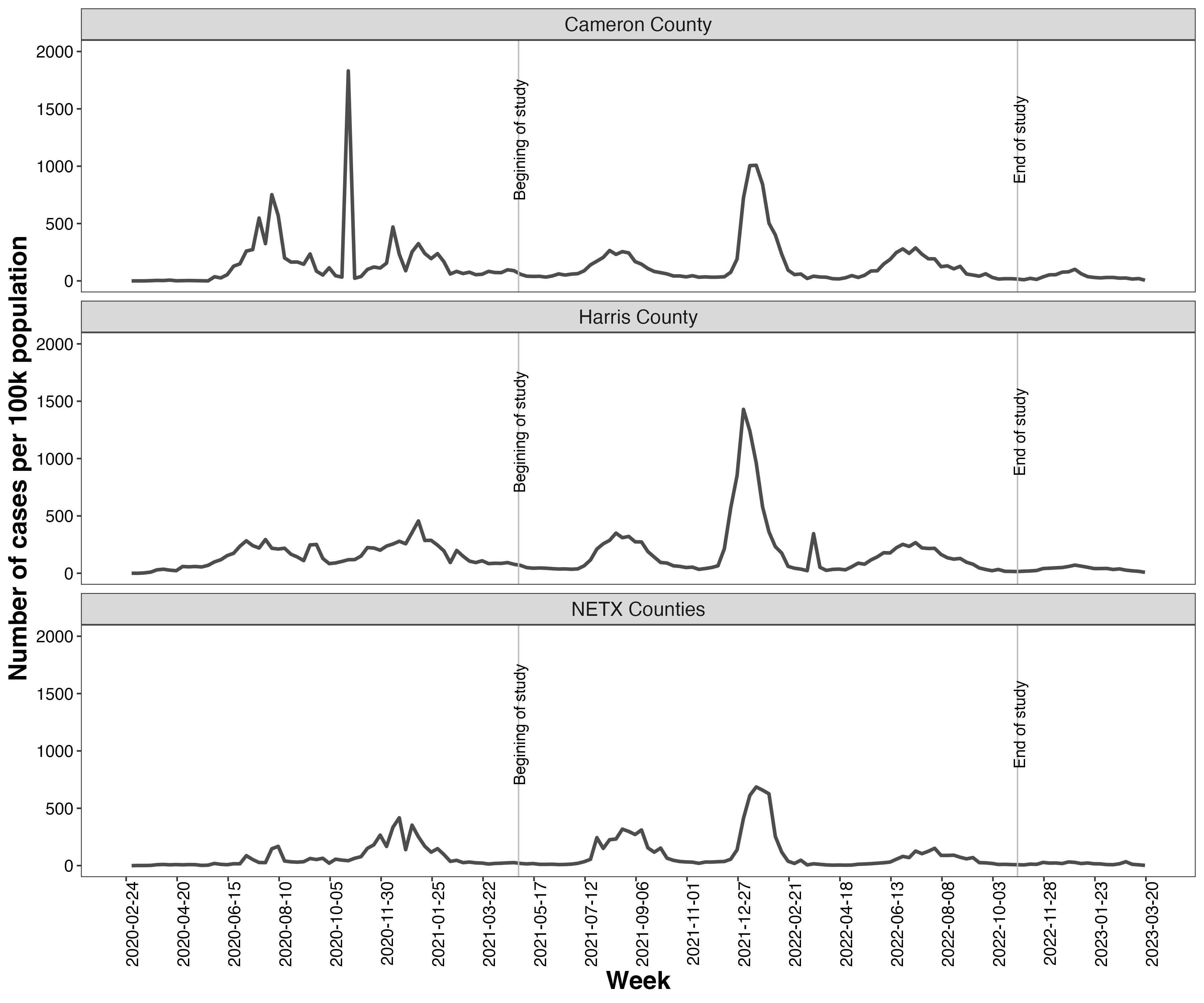

Supplement: Multimedia Appendix 3 [file formative-v9-e62802-s003.png]

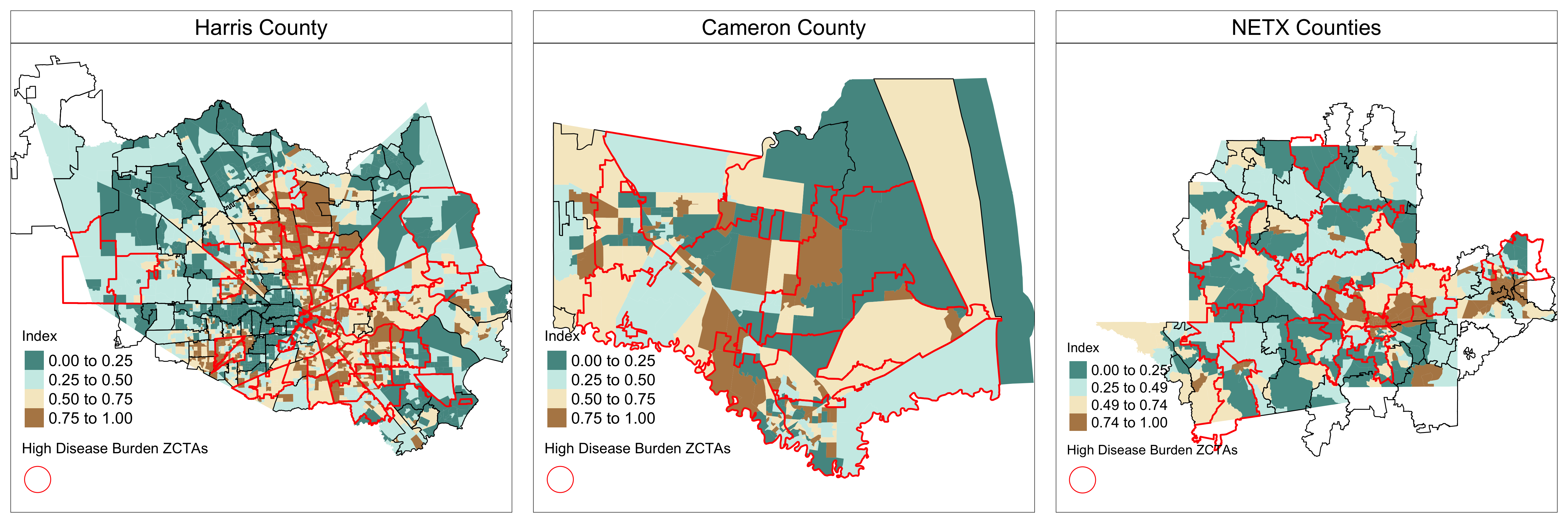

Supplement: Multimedia Appendix 4 [file formative-v9-e62802-s004.png]
