# Supplementary material for: Increasing COVID-19 Testing and Vaccination Uptake in the Take Care Texas Community-Based Randomized Trial: Adaptive Geospatial Analysis
Source: JMIR Form Res. 2025 Feb 11;9:e62802. doi: 10.2196/62802 (PMC11835599; doi:10.2196/62802)
Supplement: Multimedia Appendix 2 [file formative-v9-e62802-s002.docx]

Metrics for identifying vulnerable populations, definitions, data sources and available spatial scale.

| **Metrics** | **Definition** | **Data Source** | **Geospatial Unit** |
| --- | --- | --- | --- |
| **High disease burden** | - Persistent: number of days with ≥10 daily cases/100k from March 1^st^, 2020, to the time of analysis - Recent: number of days with ≥ 80^th^ percentile daily cases/100k in the last 30 days | Local Public Health Department Case Investigation Data | Zip Code Tabulated Areas (ZCTA) |
| **Community disparity index** | Derived metric from 12 American Community Survey (ACS) variables. | 2014-2018 5-year estimates from American Community Survey (ACS) by the U.S. Census Bureau | Census block group (CBG) |
| Unemployment | % Persons in the civilian population aged 16 years and older who are unemployed |  |  |
| Living in poverty | % Households whose income in the past 12 months is below the poverty level |  |  |
| Per capita income | Per capital income in 2018 inflation-adjusted dollars |  |  |
| No high school diploma | % Persons aged 25 years and older with no high school diploma |  |  |
| Uninsured | % Persons in the civilian noninstitutionalized population who are uninsured |  |  |
| Age under 18 | % Persons under 18 years old |  |  |
| Age over 65 | % Persons over 65 years old |  |  |
| Single-parent households | % Single-parent households with children aged less than 18 years old |  |  |
| Racial minority | % Persons who are not non-Hispanic white |  |  |
| Renters | % Occupied housing units that are renter-occupied |  |  |
| Rent burden | % Renters paying more than 30 percent of the household income for rent |  |  |
| Crowded housing | % Occupied housing units with more than one occupant per room |  |  |
