# Supplementary material for: Increasing COVID-19 Testing and Vaccination Uptake in the Take Care Texas Community-Based Randomized Trial: Adaptive Geospatial Analysis
Source: JMIR Form Res. 2025 Feb 11;9:e62802. doi: 10.2196/62802 (PMC11835599; doi:10.2196/62802)

Screenshots of the developed (A) R shiny dashboard and (B) ArcGIS dashboard developed for the Take Care Texas study. These data dashboards were designed to be interactive to best facilitate strategic planning efforts and were password-protected for internal team use only.

(A)

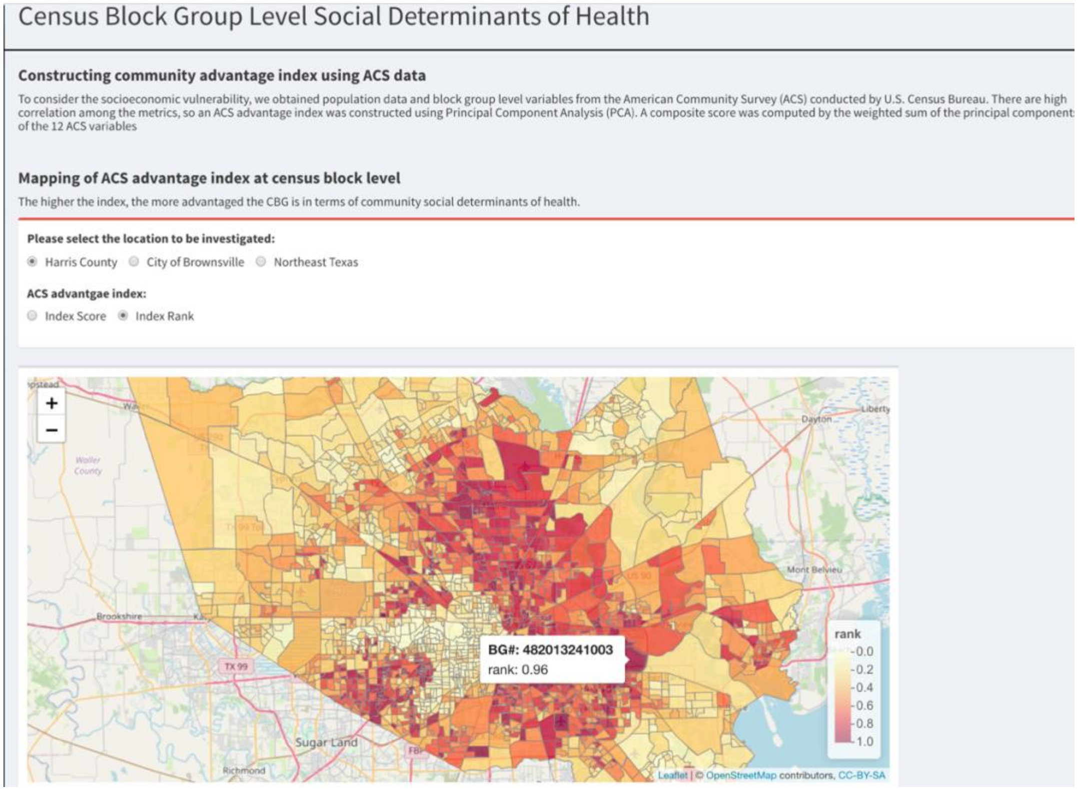

(B)

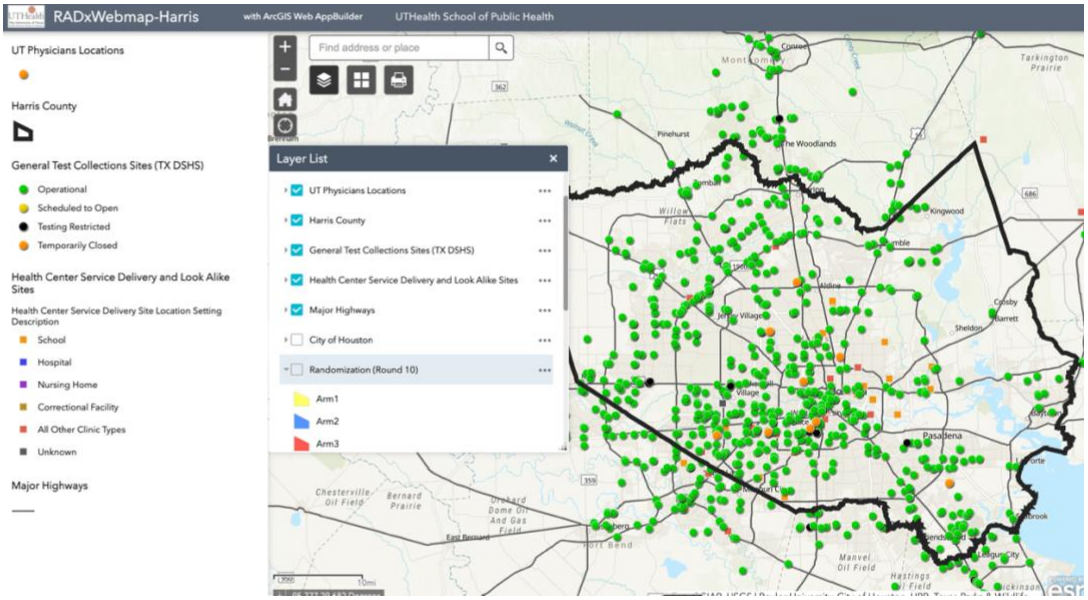

Supplement: Multimedia Appendix 5 [file formative-v9-e62802-s005.pdf]
